# BoNT/E-associated protein disrupts intestinal epithelial barrier integrity to facilitate transcytosis

**DOI:** 10.3389/fmolb.2026.1855747

**Published:** 2026-06-25

**Authors:** Sirisha V. Mukkawali, Soniya Balli, Aisha Furey, Paul Lindo, Raj Kumar, Bal Ram Singh

**Affiliations:** Botulinum Research Center (BRC), The Institute of Advanced Sciences (INADS), North Dartmouth, MA, United States

**Keywords:** bioenhancer, botulinum neurotoxin, P80, tight junctions, transcytosis, circular dichorism, internalization, transwell

## Abstract

**Background:**

Epithelial and endothelial barriers play essential roles in maintaining compartmentalization within the body through specialized intercellular junctional complexes. Botulinum neurotoxin type E (BoNT/E), a ~150 kDa protein toxin produced by *Clostridium botulinum*, is capable of traversing the intestinal epithelial barrier despite its large molecular size. BoNT/E is produced in association with several neurotoxin-associated proteins (NAPs), which are believed to facilitate toxin stability and absorption. Among these, an approximately 80 kDa protein (P80), corresponding to OrfX2, has been identified as a BoNT/E-associated protein; however, its functional role in epithelial transport remains poorly understood.

**Methods:**

P80 was purified from the BoNT/E progenitor toxin complex and characterized using circular dichroism spectroscopy and thermal denaturation analysis. Its biological activity was evaluated using hemagglutination assays, cytotoxicity studies, confocal microscopy, atomic force microscopy (AFM), immunofluorescence imaging, tight junction protein analysis, and transcytosis assays in Caco-2 and HT-29 intestinal epithelial cell models.

**Results:**

Structural analyses revealed that P80 is a stable, predominantly α-helical protein with a well-defined tertiary structure. P80 exhibited no detectable hemagglutination activity and showed minimal cytotoxicity toward intestinal epithelial cells. Cellular studies demonstrated that P80 interacts with epithelial monolayers, induces actin cytoskeletal remodeling, increases claudin-1 phosphorylation, and alters tight junction organization. AFM and confocal microscopy revealed changes consistent with transient modulation of epithelial barrier integrity. Functional transcytosis studies showed that P80 significantly enhanced the transport of co-incubated macromolecular cargo across polarized Caco-2 monolayers, resulting in approximately a fivefold increase in translocation compared with untreated controls.

**Discussion:**

These findings demonstrate that P80 functions as a non-toxic modulator of epithelial barrier permeability capable of enhancing paracellular transport of large biomolecules. Unlike classical hemagglutinin-associated neurotoxin proteins, P80 lacks hemagglutination activity while retaining the ability to reversibly alter tight junction dynamics. The results suggest that P80 contributes to BoNT/E intestinal absorption and may serve as a promising bioenhancer for improving mucosal delivery of therapeutic proteins and other hydrophilic macromolecules. Further mechanistic and *in vivo* studies are warranted to evaluate its translational potential.

## Graphical Abstract

**Figure:**
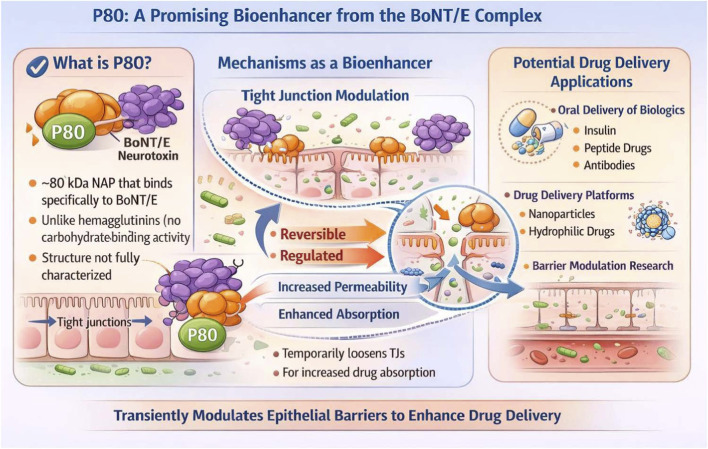

## Introduction

Botulinum neurotoxin (BoNT), produced by *Clostridium botulinum*, is the most potent toxin known to humans and the causative agent of botulism, a paralytic illness that can lead to respiratory failure and death if left untreated. BoNT belongs to a family of toxins known as A-B type toxins, characterized by a dual-domain structure. The “A” domain is responsible for the enzymatic action within the target cell, whereas the “B” domain mediates the binding and internalization of the toxin into neuronal cells. However, BoNT is produced not as a free neurotoxin but as part of a large protein complex that includes several non-toxic proteins, collectively referred to as neurotoxin-associated proteins (NAPs) (Sharma et al., 2003; Poulain et al., 2008). These NAPs play a crucial role in the stability, activity, and toxicity of BoNT. They are believed to protect the neurotoxin from digestive proteases and the acidic environment of the stomach, thereby facilitating the toxin’s survival as it traverses the gastrointestinal tract and enters systemic circulation (Somers and DasGupta, 1991; Schantz and Johnson, 1997; Ahnert-Hilger and Bigalke, 1995; Fujinaga et al., 2009). Importantly, the BoNT complex (e.g., 19S or 16S forms) is significantly more toxic than the pure neurotoxin (9S form), with the latter being about 1,000 times less potent (Zhang and Singh, 2004).

### Botulinum neurotoxin and its complex formation

Botulinum neurotoxins (BoNTs), produced by the bacterium *Clostridium botulinum*, are among the most potent neurotoxins known to science. These toxins are synthesized as large protein complexes composed not only of the neurotoxin itself but also of several associated non-toxic proteins (NAPs). These NAPs, which vary in size and function, are increasingly recognized for their crucial roles in enhancing the toxicity, stability, and biological activity of BoNT. The interplay between BoNT and NAPs is essential to the understanding of the toxin’s mechanisms, particularly how it survives hostile environments like the gastrointestinal (GI) tract and ultimately reaches its target cells in the nervous system.

BoNTs are produced by *Clostridium botulinum* in a form that includes both the neurotoxin protein and a variety of NAPs. These complexes, which are sometimes referred to as BoNT-NAP complexes, exhibit different compositions based on the BoNT serotype (A, B, C, D, E, F, G) produced by the bacterium. The NAPs, which range widely in molecular weight, contribute to a variety of important functions, including the stability of the complex, resistance to proteolysis, and the enhancement of endopeptidase activity of BoNT itself. One of the primary roles of the NAPs is to protect the neurotoxin from the proteolytic activity it encounters in the gastrointestinal tract, where BoNT must survive highly acidic conditions and digestive enzymes before it can be absorbed into the systemic circulation. For instance, studies on BoNT serotypes A and E have shown that despite the differences in the exact nature of their associated NAPs, these complexes share important properties, such as resistance to proteases and increased stability in harsh environments (Zhang and Singh, 2004; Kukreja and Singh, 2007). These characteristics allow BoNT to remain intact while passing through the acidic stomach and digestive tract, making its journey to the bloodstream more feasible.

The gastrointestinal (GI) tract presents a formidable barrier to the entry of BoNT into the body. BoNT must traverse the acidic pH of the stomach and the digestive enzymes present in the small intestine without being degraded. BoNTs, particularly those of *Clostridium botulinum* type E, have been shown to remain largely undissociated in the GI tract, even when exposed to conditions in the duodenum that are relatively neutral in pH (Sugii et al., 1977). This suggests that the NAPs associated with BoNT play an essential role in stabilizing the neurotoxin, ensuring that it is not degraded by the acidic environment or by digestive enzymes like pepsin and trypsin.

The protection afforded by NAPs appears to extend beyond simply preventing degradation. Studies have shown that the presence of NAPs contributes to the structural integrity of the BoNT complex, allowing it to remain stable through the acidic stomach and the digestive enzymes in the small intestine (Sugii et al., 1977). This stability is critical, as it allows the neurotoxin to survive long enough to be absorbed into the intestinal epithelium intact. Once the complex reaches the epithelial cells of the intestine, it can then be absorbed into the lymphatic system, from where it can enter the bloodstream and ultimately be transported to its target cells in the nervous system (Ohishi et al., 1977).

A particularly important class of NAPs involved in the BoNT complex are the hemagglutinins (HAs), which are carbohydrate-binding proteins. Heamgglutinins are known to interact with specific glycoproteins and glycolipids (Fujinaga, 2010; Kumar et al., 2014) on the surface of the intestinal epithelium, particularly enterocytes. This binding is crucial in the absorption of BoNT from the digestive tract into the bloodstream. Hemagglutinins not only protect the neurotoxin from degradation but also play an active role in the disruption of tight junctions between intestinal epithelial cells. The disruption of these tight junctions, which normally act as a barrier to the movement of molecules between cells, facilitates the translocation of BoNT across the epithelial layer into the systemic circulation (Sugawara and Fujinaga, 2011). This mechanism enhances the toxicity of the BoNT complex by increasing the amount of neurotoxin that can enter the bloodstream, thereby facilitating its eventual entry into the nervous system. Hemagglutinins also play a significant role in preventing BoNT from being flushed out of the intestinal tract, thus enabling the neurotoxin to reach the bloodstream more effectively. Recent research has focused on the molecular mechanisms by which hemagglutinins and other NAPs alter the integrity of the intestinal epithelium. These studies have demonstrated that these NAPs interact with carbohydrate receptors on the surface of enterocytes, allowing BoNT to bind tightly to the epithelial cells and promote the paracellular transport of the neurotoxin across the intestinal barrier. Additionally, these interactions may also alter the permeability of the epithelial cell layers, making them more susceptible to disruption, which further facilitates the entry of BoNT into the body (Sugawara and Fujinaga, 2011). Therfore, the ability of BoNT to enter the bloodstream is itself dependent on the action of the NAPs, which protect the toxin in the hostile environment of the digestive tract and facilitate its entry into the lymphatic and circulatory systems. The role of hemagglutinins in facilitating BoNT absorption by disrupting tight junctions between enterocytes is particularly crucial to the overall toxicity of the neurotoxin.

In the current study, we explore the role of P80, one of the NAPs associated with BoNT/E, which specifically binds to the BoNT/E neurotoxin. P80’s ability to disrupt tight junctions and its potential for enhancing toxin absorption is examined using fluorescence microscopy and AFM, techniques that allow for real-time visualization of changes in epithelial cell integrity. Additionally, structural analyses of P80 using CD, provide important insights into the protein’s conformation and its interaction with epithelial cells. These findings suggest that P80, while lacking hemagglutinin activity, may still play a crucial role in altering the integrity of the intestinal epithelial barrier, which is key for facilitating toxin absorption and contributing to the pathogenesis of botulism.

The current study provides new insights into the structural and functional roles of P80, a key NAP associated with BoNT/E. By elucidating the mechanisms by which P80 interacts with intestinal epithelial cells and alters tight junction integrity, we enhance our understanding of the molecular events that facilitate BoNT absorption. These findings not only contribute to our knowledge of botulism pathogenesis but also offer potential targets for therapeutic intervention aimed at blocking toxin entry into the host. Further studies are needed to explore the broader implications of NAPs in BoNT toxicity and their potential as therapeutic targets in botulism treatment.

## Materials

### Bacterial strain and growth conditions

*Clostridium botulinum* type E (ATCC, Manassas, VA; Alaska strain) was cultured in an anaerobic environment on trypticase-peptone glucose-sodium thioglycolate medium (BBL Microbiology Systems, Cockeysville, MD). Yeast extract, a critical component for culturing, was purchased from Difco (Sparks, MD). Glucose and ammonium sulfate, used in buffer preparations, were sourced from Research Products International Corp. (RPI, Mt. Prospect, IL). Beads for column chromatography were obtained from Pharmacia (Kalamazoo, MI).

### Cell lines and culture conditions

Two epithelial colorectal adenocarcinoma cell lines, CaCo-2 and HT-29, were purchased from American Type Culture Collection (ATCC, Manassas, VA). Both cell lines were cultured in Dubelco’s Minimum Essential Media (DMEM), supplemented with fetal bovine serum (FBS) and antibiotics, also sourced from ATCC. The cell culture flasks and plates used for maintaining and treating the cell lines were purchased from Denville Scientific (Holliston, MA).

### Cell culture materials

For all cell culture experiments, including MTT assays, 10x Radio Immunoprecipitation (RIPA) buffer, Bovine Serum Albumin (BSA), and other reagents were purchased from Sigma-Aldrich (St. Louis, MO). The MTT assay kits for cell viability measurement were also obtained from ATCC. The coverslips used for microscopy and imaging were purchased from Thermo Fisher Scientific (Pittsburgh, PA), and the polycarbonate inserts for cell migration studies were sourced from Corning (Corning, NY).

### Hemagglutination assay materials

For the hemagglutination assays, U-bottom microtiter plates were obtained from Falcon (Becton Dickinson, NJ). Alsver’s solution, used for hemagglutination, was purchased from Sigma (St. Louis, MO). Phosphate-buffered saline (PBS) and sodium chloride (NaCl), necessary for assay preparation, were also sourced from RPI (Mt. Prospect, IL).

### Fluorescence imaging and confocal microscopy

For fluorescence imaging and confocal microscopy, paraformaldehyde for cell fixation was purchased from Sigma-Aldrich (St. Louis, MO). The gold antifade mounting media for preparation of confocal slides was obtained from Thermo Fisher Scientific (Pittsburgh, PA). Fluorescein isothiocyanate (FITC)-labeled anti-actin antibody, used for cytoskeletal staining, was also purchased from Sigma-Aldrich. The secondary antibodies (anti-rabbit and anti-mouse IgG) were obtained from Sigma-Aldrich. Additionally, for imaging and microscopy, the proteinase inhibitor cocktail, essential for maintaining protein integrity during cell treatment, was sourced from Thermo Scientific (Pittsburgh, PA).

### Western blot and protein analysis

For Western blot analysis, either polyvinylidene fluoride (PVDF) membranes or Turbo Blots (BioRad, CA) and TBS and PBS buffer were purchased from Bio-Rad (Hercules, CA). The protein A/G beads used for immunoprecipitation pull-down assays were sourced from Pierce Thermo Scientific (Pittsburgh, PA). The primary antibodies used for detecting claudin (mouse anti-claudin) and actin (rabbit anti-actin) were purchased from Santa Cruz Biotechnology (Dallas, TX). For phosphoprotein analysis, anti-phospho serine/threonine/tyrosine antibody (developed in mouse) was purchased from Abcam (Cambridge, MA). Phosphatase inhibitors, sodium fluoride (NaF) and sodium orthovanadate (Na_3_VO_4_), were obtained from Roche (Indianapolis, IN). Simply Blue Safe Stain and pre-stained markers for gel analysis were purchased from BioRad (CA). Washing buffer was made from either TBS or PBS containing 0.05% tween-20 (Sigma Aldrich).

By sourcing these high-quality reagents and materials, we ensured the reliability and reproducibility of the experimental procedures. These materials enabled us to conduct a comprehensive study on the molecular interactions and structural characteristics of P80, a critical neurotoxin-associated protein (NAP) involved in the pathogenesis of botulism.

## Methods

### Protein purification

For this series of experiments, *C. botulinum* type E (ATCC Manassas, VA); Type E *Clostridium botulinum* Accession Nos. 9,564, 17,786, 17,852, 17,854, and 17,855) was grown for 4 days in 15 mL cooked meat medium. Stock cultures were prepared according to standard methods and stored at −20 C. The stock culture was activated at 30 °C. for approximately 24 h and then transferred to a growth medium containing 2.0% Trypticase-peptone, 1.0% glucose, 0.025% sodium thioglycolate (BBL Microbiology Systems, Cockeysville, MD), and 0.5% yeast extract (Difco) adjusted to pH 6.5. The culture was incubated for 70–75 h, and cells were collected by centrifugation. An extract from the cells was prepared at 20 °C by stirring with 0.2 M phosphate buffer (pH 6.0). The resulting suspension was saturated with (NH4)_2_SO_4_; 39 g/100 mL) and stored at 4 °C.

The crude extract described above was pelleted and redissolved in 35 mL of 0.05 M sodium phosphate buffer (pH 5.5). The resulting solution was clarified by centrifugation and chromatographed on a DEAE-sephadex A-50 ion-exchange column (Pharmacia). The sample was eluted from the column with 0.05 M sodium citrate at pH 5.5. It is important that the pH of the buffer is maintained at 5.5.

Eluted fraction concentration was measured by UV and pooled.

### Circular dichroism spectroscopy

The P80 protein was dialyzed in 25 mM Na-Citrate Buffer pH 6. Circular Dichroism (CD) data was collected using JASCO J-715 spectropolarimeter (Jasco Inc., Easton NJ) equipped with a Peltier temperature control (Model PTC-348 W) as described previously (Kumar and Singh, 2025). Far-UV CD spectra in the 190–250 nm wavelength region was recorded in a 1.0 mm path length cuvette with a protein concentration of 0.2 mg/mL at 25 °C at a scanning speed of 20 nm/min and with a response time of 8 s. A total of three scans were recorded and averaged to increase the signal to noise ratio. The final spectra were obtained after correcting the buffer contribution. The data presented was normalized for the concentration of proteins.

### Temperature-induced unfolding measurement

Temperature induced unfolding of P80 was followed by recording change in the CD signal at 222 nm with temperature change (Kumar and Singh, 2025). The protein samples were dialyzed in 25 mM Na-Citrate Buffer pH 6. The samples were heated in the temperature range of 25 °C–90 °C with a heating rate of 1 °C/min. The normalized mean residue ellipticity at 222 nm was plotted as a function of the temperature.

### Cell toxicity measurement

Cell viability was evaluated with mitochondrial activity. The mitochondrial activity of CaCo-2 cells was measured after treatment with different concentrations (300 nM) of P80 by the MTT (3-(4, 5-dimethylthiazol-2-yl)-2, 5-diphenyltetrazolium bromide) assay after the 24-h time point.

∼10^5^ CaCo-2 cells were seeded per well in a 96 well polystyrene plate placed in an incubator (maintained at 37 °C, 95% relative humidity, 5% CO_2_) and grown in complete medium for 24 h. For each concentration (75,150,300 nM) six different wells were used. 70% ethanol was used as a negative control.

The MTT assay was carried out by replacing the spent media with either serum free media or serum free media containing appropriate concentrations of P80, or ethanol for 24 h. 10 μL of MTT reagent was added and incubated until a purple precipitate was observed (2-4 h). Once a precipitate was observed, 100 µL of detergent was added to the well and swirled gently to mix the contents. The plate was covered and left in the dark overnight before it was read at 570 nm. The concentration of the P80 that was selected for further analysis was thus observed to have optimal mitochondrial activity similar to the control cells. The cytotoxicity studies for Hn33 were performed and reported by Kumar et al. (2012).

### Hemagglutination assay

The assay was performed in a U-bottom microtiter plates according to the procedure of DasGupta and Sugiyama (1977). Freshly drawn human blood (Type O positive) was used and treated with Alsver’s solution. Red blood cells were centrifuged at 400 rpm and washed with 0.8% NaCl (Normal Saline) three times before being used. 0.005 mL of 0.5% suspension of erythrocytes in phosphate buffered saline was added to each well. The proteins were diluted from 50 µg to 5 μg per well, by increments of 5 µg. Hemagglutination activity was considered positive when the wells did not show accumulation of erythrocytes in the middle, either in an annular shape or as a red dot. Bovine serum albumin was used as a negative control with similar dilution.

### Internalization of P80 and Hn-33 in intestinal cells

Caco2 and HT-29 cells were grown on glass coverslips and incubated with serum free media, containing AlexaFluor 488 labeled P80 and Hn-33 for varying time (2, 4, 6, 8, 24 h) in a 37 °C humidified incubator with 5% CO_2_ to determine the internalization of these proteins. After incubation, cells were washed with 1 x PBS and fixed with 4% paraformaldehyde and imaged using confocal microscope (Zeiss LSM 700 confocal microscope at core facility of Brown University). For membrane and nucleus, WGA (wheat germ agglutinin) and DAPI (4′, 6-diamidino-2-phenylindole) were used. The fluorescence intensities were averaged 5 times to get the statistically significant data.

### Immunofluorescence microscopy

For imaging experiments, CaCo-2 cells were seeded on cover glasses and grown to desired confluency in a 37 °C, 5% CO_2_ humidified incubator. The cells were treated using 150 nM of Hn33 or P80. After incubation, the cell monolayers were thoroughly washed with phosphate buffered saline, pH 7.4 (PBS) pre-warmed to 37 °C and fixed with freshly prepared 4% paraformaldehyde for 15 min at room temperature and washed twice with PBS. Then the cells were permeabilized using 0.2% Triton X-100 in blocking solution, made of 1% (w/v) bovine serum albumin (BSA) in PBS, for 20 min, so as to make the cell wall permeable. The permeabilized cells were then washed twice with PBS and incubated with 250 μL of 1% BSA blocking solution for 30 min. For immunofluorescent staining, the blocking solution was removed, and cells were incubated with 250 µL of FITC monoclonal anti-Actin raised in rabbit or anti-ZO-1 raised in rabbits for one hour at room temperature. After incubation, the cells were washed three times with PBS and mounted on a glass slide using a drop of Prolong Gold antifade mounting media (Invitrogen) and imaged using a Zeiss LSM 700 confocal microscope. The change in the pattern of actin proteins was monitored by detecting the fluorescence elicited at excitation wavelength of 490 nm on a Zeiss 710 confocal microscope. The image was averaged 16 times to result in the final image.

### Expression and purification of detoxified recombinant botulinum neurotoxin (DrBoNT)

DrBoNT was expressed and purified as described previously by Yang and colleagues (Yang et al., 2009).

### Labeling of P80, Hn-33 and DrBoNT

For transcytosis and imaging experiments, proteins were labeled with AlexaFluor-488 Molecular Probes (Thermo Fisher Scientific). All labeling followed the protocol provided by the vendor and the labeled protein was purified using the column provided by manufacturer. Protein concentrations of labeled proteins were compared to unlabeled proteins were measured and further calculated for Protein to Dye ratio.

Confocal laser scanning microscopy: For imaging experiments, CaCo-2 and HT-29 cells were seeded on cover glasses and grown to desired confluency in a 37 °C, 5% CO_2_ humidified incubator. The protocols were according to earlier published work (Kumar et al., 2012). The cells were treated using 150 nM of Hn33 or P80 and incubated for different time points. After incubation, the cell monolayers were thoroughly washed with phosphate buffered saline, pH 7.4 (PBS) pre-warmed to 37 °C and fixed with freshly prepared 4% paraformaldehyde for 15 min at room temperature and washed twice with PBS. Then the cells were permeabilized using 0.2% Triton X-100 in blocking solution, made of 1% (w/v) bovine serum albumin (BSA) in PBS, for 20 min, so as to make the cell wall permeable. The permeabilized cells were then washed twice with PBS and incubated with 250 μL of 1% BSA blocking solution for 30 min. For immunofluorescent staining, the blocking solution was removed, and cells were incubated with 250 µL of FITC monoclonal anti-Actin raised in rabbit or anti-ZO-1 raised in rabbits for one hour at room temperature. For P80 and Hn-33 localization experiment, alexa-fluor 488 labeled P80 and Hn-33 were used (Dye:Protein::2-3:1) for permeabilization. For membrane and nucleus staining WGA and DAPI were used, respectively. After incubation, the cells were washed three times with PBS and mounted on a glass slide using a drop of Prolong Gold antifade mounting media (Invitrogen) and imaged using a Zeiss LSM 700 confocal microscope. The change in the pattern of different proteins was monitored by detecting the fluorescence elicited at excitation wavelength of 490 nm on a Zeiss 710 confocal microscope. The image was averaged 16 times to result in the final image.

### Atomic force microscopy

*Cell Monolayer Preparation:* Human colorectal adenocarcinoma cells (CaCo2) were routinely grown and maintained in T25 flasks in EMEM media with 20% FBS media supplemented with 20% fetal bovine serum (FBS), in an incubator (maintained at 37 °C, 95% relative humidity, 5% CO2). For experiments, cells were seeded on a coverslip in a flat-bottomed cell culture plate and grown to form a monolayer.

*Treatment of cells with P80 or Hn-33:* Media from the wells was removed & discarded. The cells were washed twice with serum free media & incubated with the respective serum free media containing P80 or Hn33 at a concentration of 150 nM for 2 h in an n incubator (37 °C, 95% relative humidity, 5% CO2). After the incubation period, the cells were washed three times with PBS. The samples were then dried out in the hood for 30 min before being used for AFM analysis.

*Imaging:* The AFM imaging was carried out using non-contact mode, with a cantilever having a spring constant of 42 N/m, on a Pak Systems XE 100 atomic force microscope. Several different scan sizes were identified to obtain representative images of cells with and without P80 or Hn33 treatment. Later, these images were analyzed using image analysis software provided by the manufacturer of the AFM.

### Transcytosis of labelled DrBoNT/A through polarized CaCo-2 monolayers

Both CaCo-2 and HT-29 cells were grown to 80% confluence and sub-cultured in DMEM supplemented with 10% FBS and 1% Pen-Strep and Non-essential amino acids solution respectively. All experiments in this study were performed on CaCo-2 cells between 4–15 passage levels.

For transcytosis experiments with CaCo-2 and HT-29, cells were trypsinized using Trypsin-EDTA, re-suspended (5 × 10^5^ cells/mL) in DMEM, and then added (500 µL) to polycarbonate inserts, 12 mm transwell, 24 well plate with 0.4 μm membrane insert (Corning Transwell, Corning, NY). Media in the apical and basolateral sides was changed every 2 days. In case of CaCo-2 cells, an intact cell monolayer was formed by day 10. Whereas in case of HT-29, an intact cell monolayer was formed by day 5. Integrity of the cell monolayer was also confirmed by microscopic evaluation of the cell monolayer and monitoring the level of the medium in the apical chamber (Von Bonsdorff et al., 1985). On day 10, apical medium was supplemented with 200 nM of Alexa-488 labelled DrBoNT/A (negative control sample). In case of the test sample, a mixture of 200 nM of Alexa-488 labelled DrBoNT/A and 150 nM of P80 or Hn33 was supplemented to the apical side of the inserts. The cells incubated at 37 °C, 5% CO2. 500 μL of serum free media was also added to the basolateral side of the insert. The plates were incubated at 37 °C, 95% relative humidity, 5% CO_2_, at designated time points, 60 µL aliquots were removed from the basal medium, placed in a black microtiter plate, and fluorescence was read at 525 nm with excitation at 490 nm.

### Pull down and immunoprecipitation assay

Immunoprecipitation assay for claudin phosphorylation pulldown: After incubation with P80 and Hn-33, the cells were washed three times with cold PBS, scraped and pelleted down. For cell lysis of 50 mg cell pellet, we used 500 µL of cold lysis buffer (25 mM Tris-HCl, pH 7.4, 150 mM NaCl, 1% Triton X-100, 1 mM EDTA, 5% glycerol, 1 mM NaF, 1 mM Na_3_VO_4_, protease inhibitor, phosphatase inhibitor cocktail (Invitrogen), incubated with mixing intermittently at 4 °C for 20 min. Insoluble material was removed by centrifugation at 10,000 *g* for 20 min at 4 °C. Soluble material was analyzed by immunoblot or used for immunoprecipitation. Phosphorylated proteins were immunoprecipitated with monoclonal anti-phosphoserine/threonine/tyrosine antibody developed in mouse (Abcam) 10 µL of antibody (1 mg/mL conc.)/50 mg of cell pellet, overnight incubation). Immunocomplexes were captured using 20 µL agarose conjugated protein A/G beads (Pierce) by gently rotating at 4 °C for 2 h and then washed three times with 200 µL of cold lysis buffer, including phosphatase and protease inhibitors at 4 °C. Final wash was done with 1X saline solution (150 mM NaCl). Immunoprecipitates were dissolved in 50 µL of 2 X SDS-PAGE (Sodium Dodecyl Sulfate Polyacrylamide Gel Electrophoresis) reducing sample buffers, boiled for 10 min, and analyzed by SDS-PAGE and immunoblotting. For immunoblots, protein bands were electro transferred onto PVDF membranes, blocked with 5% bovine serum albumin (BSA) in TBST, and immunoblotted using anti-claudin antibody raised in mouse (1:2,500 in 3% BSA-TBST). Anti-mouse IgG alkaline phosphate conjugate antibodies (Santa Cruz Biotechnology) were used as secondary antibodies. The Bands were visualized using final using alkaline phosphatase substrate NBT/BCIP (Sigma).

## Results

P80 was found to be to one of neurotoxin associated protein that had the ability to directly bind to the BoNT/E toxin. To further understand the biological activity of P80, a preliminary knowledge about its structure is essential.

### Expression and purification of P80

**FIGURE 1:**
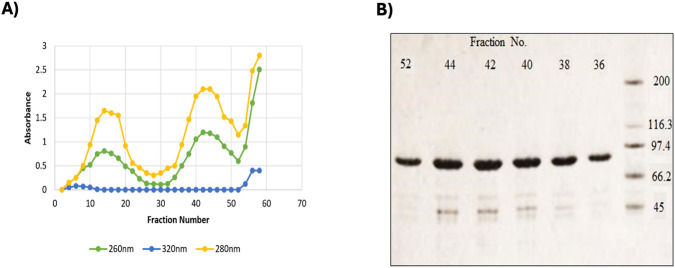
Elution profile of DEAE column and SDS-PAGE analysis of selected fractions. **(A)** The DEAE anion-exchange chromatography was performed to separate the components of the sample. The elution profile shows two distinct peaks: Peak 1 corresponds to the Botulinum Neurotoxin E (BoNT/E) L-chain complex, while Peak 2 corresponds to the P80 protein. **(B)** Selected fractions from the DEAE elution profile were analyzed by SDS-PAGE to confirm the identity and purity of the separated proteins. Fractions from Peak 2 display a band correspond to the P80 protein.

In the current work, the protein, P80, was purified from the BoNT Type E complex in two steps, using DEAE sephandex A-50 anion exchange column followed by CM sepharose CL-6B (Cytiva, United States) cation exchange column. All the purification steps were performed at 4 °C. Notably, the CM sepharose column resulted in pure P80 fraction. An elution profile of different fractions from the DEAE sephadex A50 column (Cytiva, United States) is shown in Figure 1. The first protein peak seen in the elution profile was pooled as Type/E L complex. The second protein peak was analyzed by running an SDS gel of selected fractions (36, 38, 40, 44, and 52) from the column. The fractions obtained were tested on SDS-PAGE for purity (Laemmli, 1970). The gel (Figure 1B) showed that these fractions contained primarily P80 protein with some impurities. Eluted fraction concentration was measured by UV and pooled. Pooled P80 fractions were then loaded on CM Speharose CL-6B (cation exchange) column pre equilibrated with buffer (20 mM Sodium Citrate buffer, pH 6.0). As the pI of P80 was found to be ∼6.3 (as calculated using online available software http://isoelectric.ovh.org/), the protein does not bind to the column due to its pI equivalent to buffer pH and hence is eluted. The ion exchange column was used to separate protein from impurities based on charge. The purified P80 was stored in 20% glycerol solution at −80 °C after flash freezing in liquid nitrogen till further use.

### Structural characterization of P80 protein

**FIGURE 2:**
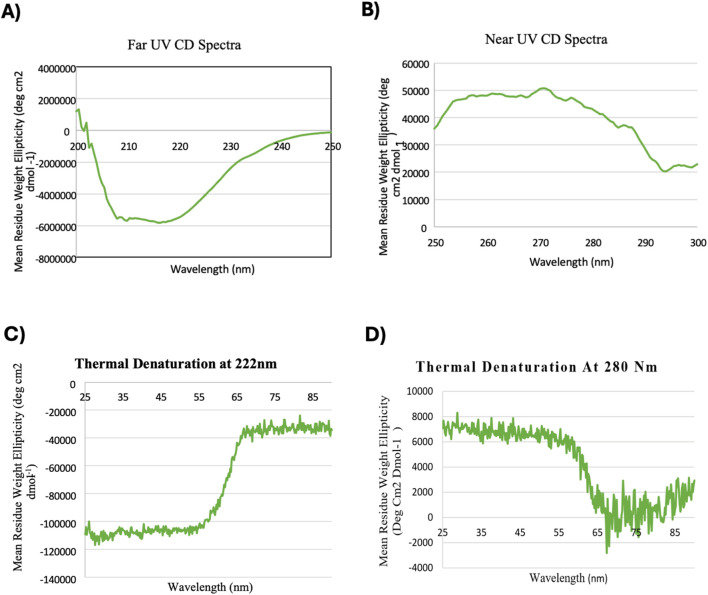
Structural analysis of P80. **(A)** *Far-UV CD Spectrum of P80:* The far-UV circular dichroism (CD) spectrum of P80, recorded at pH 6, reveals a characteristic double minimum around 208 nm and 220 nm, indicative of an α-helical structure with contributions from random coils. **(B)** *Near-UV CD Spectrum of P80:* The near-UV CD spectrum of P80 shows distinct peaks corresponding to aromatic side chains, with contributions from tyrosine (Tyr), tryptophan (Trp), and phenylalanine (Phe) residues. A minimum is observed at 280 nm, consistent with the asymmetry around the tyrosine residues. Additional minima are observed at approximately 285 and 295 nm, likely due to the aromatic side chains of Tyr and/or Trp. The spectrum indicates a well-defined tertiary structure with strong near-UV signals characteristic of a folded protein. **(C)** *Thermal Denaturation of Secondary Structure* (222 nm)*:* The thermal denaturation profile of P80, monitored by CD at 222 nm (far-UV), shows a sigmoidal transition as the protein unfolds upon heating. **(D)** *Thermal Denaturation of Tertiary Structure* (280 nm): The thermal denaturation profile of P80, recorded at 280 nm (near-UV), reveals a similar sigmoidal unfolding transition, beginning at around 55.8 °C and ending at 64.4 °C.

The secondary structure of globular proteins can be probed using circular dichroism (CD) spectroscopy, which offers insights into the conformational states of the protein. In this study, both far-UV and near-UV CD spectra were recorded to evaluate the structural features of P80 (Figures 2A,B). The far-UV CD spectrum, which provides information on the secondary structure, revealed a typical pattern representative of an ensemble of the protein’s molecular population. At pH 6, the spectrum displayed a characteristic double minimum around 208 nm and 220 nm, indicative of an α-helical structure, with some contributions from random coil regions. This suggests that P80 adopts a predominantly α-helical conformation with some flexible, disordered regions. The mean residue ellipticity at 222 nm for P80 was measured at −5,635,920 degcm^2^·dmol^-1^ (Figure 2A), while at 208 nm, it was slightly less intense, registering −5,564,360 deg cm^2^·dmol^-1^ (Figure 2A). The ratio of ellipticities at 222 nm–208 nm (θ_o222_/θ_o208_ > 1) further supports the dominance of an α-helical structure with coiled-coil characteristics, which is a typical feature of proteins with a compact, structured fold.

Near-UV CD spectroscopy, which provides insight into the tertiary structure of proteins by probing the aromatic side chains and disulfide bonds, further supported the conclusion that P80 is a well-folded protein. The presence of strong near-UV signals is indicative of a stable, folded tertiary structure. The amino acid composition of P80 includes approximately 10% aromatic residues, including 24 tyrosine (Tyr), 35 phenylalanine (Phe), and 12 tryptophan (Trp). The near-UV CD spectrum of P80 (Figure 2B) showed a broad, positive signal with distinct peaks at the typical wavelengths for aromatic amino acid side chains, with notable contributions from tyrosine, tryptophan, and phenylalanine. A minimum was observed at 280 nm, corresponding to the absorption features associated with tyrosine and tryptophan residues. Two additional minima were observed at approximately 285 and 295 nm, likely due to the contributions of the tyrosine and/or tryptophan side chains. However, the phenylalanine region did not display as well-defined peaks, possibly due to overlapping or weak signals in this region.

To further investigate the stability of P80’s secondary and tertiary structures, we performed thermal denaturation experiments, monitoring changes in the CD signal at 222 nm (far-UV) for secondary structure and 280 nm (near-UV) for tertiary structure as a function of temperature (Figures 2C,D). The thermal denaturation curve obtained at 222 nm (far-UV) exhibited a reverse sigmoidal shape, indicative of a cooperative unfolding transition. P80 began to unfold at approximately 55.6 °C, with a steep transition occurring between 55.6 °C and 66.8 °C. The melting temperature (T_m_) of the protein, determined from the midpoint of this transition, was found to be 62.37 °C ± 0.06 °C (Figure 2C). This transition suggests that the secondary structure of P80 is highly stable, and at physiological temperature (37 °C), the protein maintains its native fold. Similar thermal unfolding experiments were performed at 280 nm (near-UV) to monitor the stability of the tertiary structure. The unfolding curve revealed a transition that began at approximately 55.8 °C and completed by 64.4 °C. The melting temperature (T_m_) for the tertiary structure was calculated to be 63.11 °C ± 0.18 °C (Figure 2D). This transition was also sigmoidal, confirming that the protein undergoes a cooperative unfolding process. Both secondary and tertiary structure thermal denaturation profiles displayed highly cooperative unfolding transitions, suggesting that P80 exists as a compact, well-folded structure under native conditions. The similarity in the melting temperatures observed for both secondary and tertiary structure unfolding (62.37 °C vs. 63.11 °C) further supports the idea of a stable, well-integrated protein structure where the folding of secondary and tertiary elements are tightly coupled.

### Evaluation of P80 toxicity and hemagglutination activity

**FIGURE 3:**
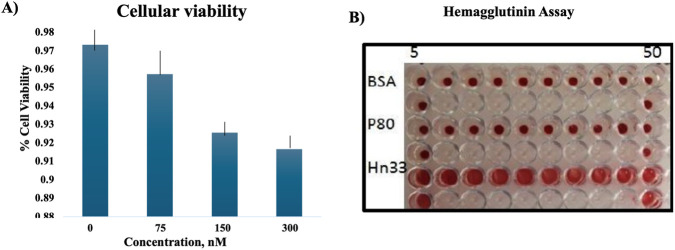
Evaluation of Cellular Toxicity and Hemagglutinin Activity of P80. **(A)** *Cellular Toxicity of P80:* Plot of absorbance values from the MTT assay, reflecting cellular viability, as a function of P80 concentration (75 nM, 150 nM, and 300 nM) after 24 h of incubation with CaCo-2 intestinal epithelial cells. The absorbance at 570 nm is proportional to the mitochondrial activity of viable cells. Data represent the mean ± standard deviation (n = 3). **(B)** *Hemagglutinin Activity of P80:* Hemagglutination assay comparing P80 with Hn33 (a positive control protein known for its hemagglutinin activity). After serial dilution of P80, Hn33 and BSA (5 μg–50 μg), standardized RBCs were added to each well and incubated for 30 min at room temperature. The presence or absence of RBC agglutination was visually assessed.

*Cytotoxicity Assay (MTT Assay*: The *in vitro* cytotoxicity of P80 was assessed using an MTT assay to measure the viability of CaCo-2 intestinal epithelial cell lines after treatment with P80 at various concentrations (75, 150, and 300 µM) for 24 h. The MTT assay determines mitochondrial activity, which correlates with the number of viable cells, by monitoring absorbance at 570 nm. All measurements were conducted in triplicate (n = 3), and the results are presented as the mean ± standard deviation. Higher absorbance values indicate greater cell viability. At concentrations of 75 μM, 150 μM, and 300 μM, the absorbance values were 0.957 ± 0.029, 0.930 ± 0.016, and 0.920 ± 0.0208, respectively (Figure 3A). These data suggest that P80 does not exhibit significant toxicity at these concentrations in the CaCo-2 cell line, indicating that P80 is unlikely to be toxic to mammalian cells at physiological concentrations.

*Hemagglutination Activity of P80*: Hemagglutination activity was used to assess the ability of P80 to bind to surface molecules on red blood cells (RBCs). The assay was performed by serially diluting P80 protein in a 96-well U-bottom plate, followed by the addition of a standardized concentration of RBCs to each well. The plate was incubated for 30 min at room temperature, and the agglutination pattern was assessed by examining the RBCs for the formation of a lattice structure. A positive agglutination result was indicated by a clear, uniform agglutinated lattice, while a negative result was characterized by a red pellet (button) formed at the bottom of the well due to the settling of non-agglutinated RBCs. As a positive control, Hn-33 (a complex protein of botulinum neurotoxin type A with known hemagglutination activity) caused agglutination even at concentrations as low as 5 μg, confirming the reliability of the assay. In contrast, the negative control, Bovine Serum Albumin (BSA) (Thermo Fisher Scientific), did not induce agglutination at any concentration, even at the highest concentration tested (50 µg). P80, similar to BSA, did not induce agglutination at any concentration tested. Instead, a dark red pellet was observed at the bottom of the well, indicating the absence of agglutinated RBCs. This result suggests that P80 does not exhibit hemagglutination activity, as evidenced by the lack of a lattice structure and the formation of the RBC pellet (Figure 3B).

### Differential interactions of P80 with intestinal epithelial cells and its effect on transcytosis

To investigate the interaction of P80 with intestinal epithelial cells, we incubated 150 nM of Alexa Fluor 488-labeled P80 with two distinct intestinal epithelial cell models, Caco-2 and HT-29, and captured fluorescence images over time using a confocal microscope. These two human colon adenocarcinoma-derived cell lines were chosen for their contrasting morphological and functional characteristics, which provide valuable insights into how P80 may interact differently with various regions of the intestinal epithelium.

Caco-2 cells are known to form tightly connected monolayers that closely resemble the intestinal epithelial layer, with prominent microvilli on the apical surface, making them particularly suited for studying absorption and permeability mechanisms. In contrast, HT-29 cells exhibit a more heterogeneous morphology with a mucus-producing phenotype and less robust tight junctions. These differences are important because they impact the transport and diffusion of molecules across the monolayer. Additionally, Caco-2 cells predominantly model absorption in the proximal colon, whereas HT-29 cells represent more distal regions of the colon and are involved in mucus secretion, inflammation, and immune cell interactions. Therefore, comparing the behaviour of P80 in these two cell models helps provide a more comprehensive understanding of its effects on the entire small intestine and distal regions.

**FIGURE 4:**
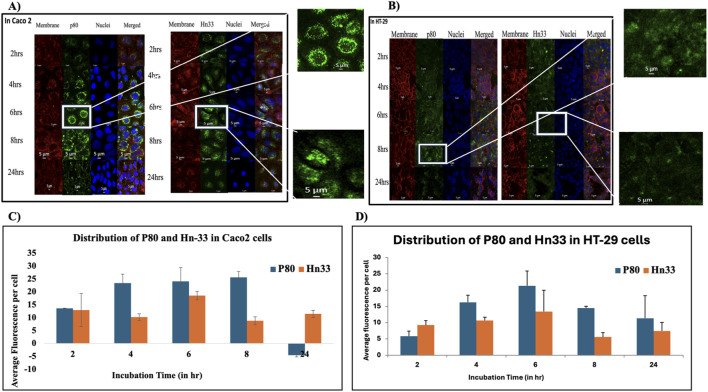
**(A)** Caco2 cells were fixed and imaged at different time points to visualize the localization of labeled P80 and Hn-33. The figure shows representative images for membrane labeling (red), P80 (green), nuclear staining (blue), and the merged image for both P80 and Hn-33. **(B)** Similarly, HT-29 cells were fixed and imaged under the same conditions, showing the labeled membrane, P80, nuclear staining, and the merged image for both P80 and Hn-33. **(C)** Quantification of internalization: Average fluorescence intensity per cell over time is plotted for P80 and Hn-33 in Caco2 cells. **(D)** Internalization kinetics: Average fluorescence intensity per cell over time is plotted for P80 and Hn-33 in HT-29 cells. The enlarged images of cellular localization of P80 and Hn-33 in Caco2 and HT-29 cells is shown (inset).

Upon incubation with 150 nM of either P80 or Hn-33, we observed a differential pattern of internalization in both cell lines over time. In Caco-2 cells, both P80 and Hn-33 attached to the cell surface rapidly, but their intracellular distribution diverged. P80 remained predominantly near the plasma membrane (Figure 4A), suggesting that it may interact more transiently with the epithelial barrier or be retained at the surface for a longer period. In contrast, Hn-33 showed a stronger tendency to internalize, becoming distributed more evenly throughout the cytoplasm. This pattern was reversed in HT-29 cells (Figure 4B), where P80 exhibited greater internalization, accumulating in the cytosol, while Hn-33 remained largely localized at the plasma membrane.

Quantification of fluorescence intensity revealed that P80 showed a significantly higher average fluorescence signal in both Caco-2 and HT-29 cells, indicating that P80 is internalized more efficiently compared to Hn-33 (Figures 4C,D). This suggests that P80 may have enhanced cellular uptake or retention capabilities compared to Hn-33 in these epithelial cell models.

These differential internalization patterns highlight the potential for exploiting the distinct interactions of P80 and Hn-33 with intestinal epithelial cells for targeted delivery across the intestinal barrier. The fact that P80 shows more efficient internalization, particularly in HT-29 cells, could indicate its suitability for crossing the epithelial layer and reaching deeper intestinal tissues. This feature might also provide an advantage in designing therapeutics aimed at crossing the intestinal epithelium.

**FIGURE 5:**
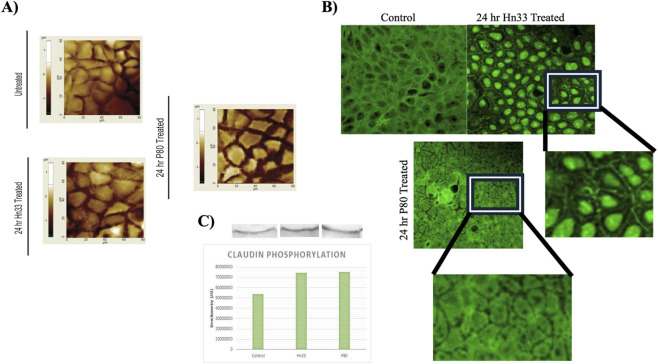
**(A)** *AFM Topography Imaging of Caco2 Cells:* Atomic force microscopy (AFM) images of Caco2 cells were acquired in non-contact mode using a cantilever with a spring constant of 0.01 N/m. Shown are images of untreated Caco2 cells, Caco2 cell monolayer after 24-h treatment with 150 nM P80, and Caco2 cell monolayer after 24-h treatment with 150 nM Hn-33. The imaged sections were 80 μm × 80 μm for both treated and untreated cells. **(B)** *FITC-Actin labeling of Caco2 Cells Treated with Hn-33 and P80*: Caco2 cells were treated with 150 nM Hn-33 or 150 nM P80 for 24 h at 37 °C and then fixed and stained with FITC-labeled actin antibody (green). The images were captured using confocal immunofluorescence microscopy with a 63x oil immersion objective. Fluorescence was detected at the 448 nm excitation wavelength for FITC on a Zeiss 710 confocal microscope. Enlarge image of actine reorganization is shown (inset). **(C)** *Effect of Hn-33 and P80 on Claudin-1 Phosphorylation*: Immunoblotting was performed using an anti-phospho serine/threonine/tyrosine antibody to assess Claudin-1 phosphorylation. Lane 1 represents the phosphorylation of Claudin-1 in control cells, lane 2 shows Claudin-1 phosphorylation after Hn-33 treatment, and lane 3 shows Claudin-1 phosphorylation after P80 treatment.

To further investigate the differential effects of P80 and Hn-33 on Caco-2 cells, both internal and external cellular changes were monitored. For internal changes, actin reorganization was analyzed using immunofluorescence, while external morphological changes were assessed using Atomic Force Microscopy (AFM), which allowed for high-resolution topographical imaging of the cell monolayer. This approach provided valuable insights into the alterations in cell morphology and structural integrity that may result from protein treatment. AFM analysis revealed notable differences in the topography of treated versus control Caco-2 cells, reflecting changes in the monolayer’s architecture. In the case of the negative control cells (Figure 5A), the monolayer maintained a tight, honeycomb-like structure typical of healthy, well-organized epithelial cells. The cells appeared closely packed with distinct cell boundaries, indicative of intact tight junctions and a well-established cellular architecture. In contrast, Caco-2 cells treated with 150 nM of Hn-33 for 24 h (Figure 5A) exhibited mild morphological changes. Although the overall packing of the cells was mostly preserved, slight striations appeared between adjacent cells, indicating subtle alterations in cell-cell interactions. This suggests that Hn-33 may induce minor reorganization or destabilization of the tight junctions, but does not lead to a significant disruption of the extrinsic environment of the cell monolayer. However, Caco-2 cells treated with 150 nM P80 for 24 h (Figure 5A) showed a more dramatic alteration in morphology. The cells no longer retained the tight packing observed in the control monolayer. There was a clear increase in the number of striations between cells, reflecting a more significant disruption of the cell-cell contacts. The increased separation between the cells suggests that P80 might be affecting the integrity of the tight junctions and promoting cellular rearrangements that lead to loosening of the monolayer. This finding is consistent with the localization data, which indicated that P80 predominantly accumulates near the plasma membrane. The accumulation of P80 at the plasma membrane may disrupt tight junctions, increasing paracellular permeability and facilitating the transport of molecules between cells. These morphological changes observed by AFM analysis align with our hypothesis that P80 induces structural alterations in the cell monolayer, potentially increasing paracellular transport. The increased separation of cells and disruption of tight junctions seen in P80-treated Caco-2 cells might enhance the paracellular pathway for molecule transport. This offers a visual confirmation of the dynamic changes that occur over time at the tight junctions, highlighting how P80 may influence epithelial barrier function and permeability.

Together, these findings suggest that while Hn-33 may cause minor changes to the morphology of the epithelial monolayer, P80 appears to significantly impact the structural integrity of tight junctions, leading to increased intercellular separation. These changes could facilitate enhanced transport across the epithelial barrier, offering important insights into how P80 may be utilized in drug delivery or therapeutics aimed at modulating epithelial permeability. The regulation of tight junction via reorganization of the actin cytoskeleton is known to be mediated via numerous signaling molecules. As the proteomic analysis revealed several proteins that appear to alter the actin cytoskeleton, confocal analysis was carried out to visualize the changes in the actin pattern.

To evaluate the effects of P80 on tight junction (TJ) permeability in human intestinal epithelial cells and compare this with Hn-33, we conducted immunofluorescence assays. These assays also allowed us to assess internal changes induced by both proteins. Caco-2 cells were exposed to 150 nM of either Hn-33 or P80 for 24 h, and actin cytoskeleton dynamics were visualized using FITC-labeled actin, following staining with actin-specific monoclonal antibodies. In control Caco-2 cells, the actin cytoskeleton appears closely associated with the plasma membrane, forming a dense band of filaments just beneath the tight junctions (Figure 5A). A small amount of actin was also observed in the cell cytoplasm, around the organelles, which appear as darker regions within the cell. This typical distribution of actin reflects the stable organization of tight junctions in healthy, untreated epithelial cells. Upon treatment with 150 nM Hn-33 for 24 h, we observed a significant reorganization of the actin cytoskeleton (Figure 5B). Actin filaments were redistributed towards the center of the cell, but interestingly, the dark regions of separation (indicative of tight junctions) remained diffused around the periphery of the cell, indicating that tight junctions were still present but had become more loosely organized. This suggests that Hn-33 induces a rearrangement of the actin network, potentially altering the structural integrity of the tight junctions, although the junctions were not entirely disrupted. Partial loss of tight junction integrity may be contributing to the changes in permeability observed with Hn-33 treatment. In contrast, Caco-2 cells treated with P80 for 24 (Figure 5C) showed less dramatic internal reorganization of the actin cytoskeleton. Although some actin rearrangement was observed, it was less pronounced than in Hn-33 treated cells. Actin appeared to shift slightly towards the center of the cell, but the overall organization was less disrupted. Interestingly, at the outer periphery of the cells, clear black cellular boundaries became visible between adjacent cells, suggesting that P80 may have a more subtle effect on tight junctions. These observations indicate that P80 induces a less severe alteration of actin organization compared to Hn-33, which could reflect a more moderate effect on the epithelial barrier. To further explore the effects of these proteins on tight junctions, we examined the phosphorylation status of claudin-1, a key scaffolding protein of the zonula occludins (ZO) family, which plays a critical role in the organization and function of tight junctions. Claudin-1 phosphorylation is known to regulate its interaction with other claudins and affect the structural stability of tight junctions. Abnormal phosphorylation, either an increase or decrease, can alter claudin aggregation and affect the permeability of the epithelial barrier. Analysis of the phosphorylation of claudin-1 using immunoprecipitation followed by Western blotting with anti-phospho-serine/threonine/tyrosine antibodies reveals the increase in phosphorylated of claudin-1 in both Hn-33 and P80 treated cells (compare to control; Figure 5C). Densitometric analysis revealed that the extent of claudin-1 phosphorylation in both treated groups was 1.38 times higher than in control cells. This increase in phosphorylation suggests that both Hn-33 and P80 may be inducing changes in claudin-1 function, potentially altering the structural integrity of tight junctions and thereby affecting epithelial barrier function. The increase in claudin phosphorylation observed in both Hn-33 and P80-treated cells may contribute to the observed changes in epithelial permeability. Enhanced claudin phosphorylation could result in modified interactions between claudin-1 and other tight junction proteins (e.g., TJP-1 (data not shown)), leading to altered junctional assembly and increased paracellular permeability. This change in barrier function could have important implications for the transport of molecules across the intestinal epithelium, potentially enhancing the absorption or translocation of therapeutic agents or, in the case of pathogens, facilitating their entry into the body.

In summary, our data suggest that both P80 and Hn-33 induce internal reorganization of the actin cytoskeleton and increase phosphorylation of claudin-1, leading to altered tight junction function and potentially increased permeability of the epithelial barrier. While both proteins appear to modulate barrier integrity, the extent and nature of these changes differ, with P80 inducing more subtle, localized effects compared to the more pronounced alterations observed with Hn-33 treatment. A few primary intracellular molecular mechanisms are typically associated with regulating barrier function of the epithelial tight junction barrier. These mechanisms include:

1. Reorganization of internal and external molecules,
2. Reorganization of proteins associated with tight junction or cytoskeleton elements

### Role of P80 in transcytosis

**FIGURE 6:**
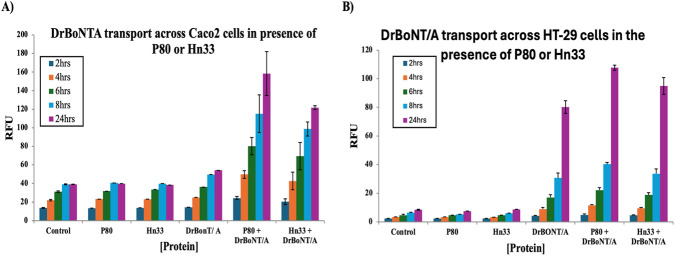
Transport of DrBoNT/A across Caco2 and HT-29 Cells. **(A)** Caco2 Cells: The transport of Alexa Fluor-488 labeled DrBoNT/A across Caco2 cell monolayers was assessed by incubating the labeled protein with P80 or Hn-33 at the apical layer. Fluorescence from DrBoNT/A in the basal solution was recorded to evaluate the transport of the protein across the cell monolayer. **(B)** HT-29 Cells: The same experimental setup was used with HT-29 cells. Alexa Fluor-488 labeled DrBoNT/A was incubated with P80 or Hn-33 at the apical layer, and the fluorescence of DrBoNT/A in the basal solution was monitored to determine its transport across the HT-29 cell monolayer.

To examine paracellular transport, we conducted a study based on previous investigations where Botulinum neurotoxin A (BoNT/A) was shown to cross epithelial cell monolayers via transcytosis (Ahsan et al., 2005; Maksymowych and Simpson, 2004; Maksymowych and Simpson, 1998). In our study, we used two intestinal epithelial cell models, Caco-2 and HT-29, to assess the differential effects of paracellular transport and the impact of P80 and Hn-33 on this process. Our findings revealed that DrBoNT/A crossed Caco-2 cell monolayers, likely via the paracellular pathway. The amount of DrBoNT/A detected in the basal medium was time-dependent, suggesting a gradual increase in translocation over time. In these experiments, a constant amount of DrBoNT/A-488 was mixed with 150 nM of P80 or Hn-33 and added to the apical side of polarized Caco-2 cell monolayers. Hn-33 was included as a positive control, as it is well-known for altering the trafficking of BoNT toxins across epithelial barriers. At designated time points (2, 4, 6, 8, and 24 h), aliquots of the basal medium were collected and analyzed for fluorescence. As shown in Figure 6A, the amount of DrBoNT/A-488 detected in the basal medium increased in a time-dependent manner. Both control and treated cells showed a steady increase in paracellular transport over time. Notably, the amount of DrBoNT/A detected in the basal medium of the Hn-33 and P80-treated cells was consistently greater than in the untreated control cells at all time points. At the 24-h time point, the translocation in P80-treated cells was approximately ∼5 fold more than in untreated controls, suggesting that P80 not only enhances the paracellular transport of DrBoNT/A but does so to a similar extent as Hn-33 in promoting the trafficking of this marker molecule across the epithelial barrier. Statistical analysis revealed no significant difference between DrBoNT/A transport in the presence of P80 or Hn-33 (p > 0.05), but transport in the presence of either protein was significantly higher (p < 0.001) compared to untreated cells.

These findings indicate that both P80 and Hn-33 modulate the rate of paracellular transport in Caco-2 cell monolayers, with P80 enhancing translocation to a comparable extent as Hn-33. The experiments provide valuable insights into the trafficking of marker molecules across epithelial barriers, highlighting that both proteins influence epithelial permeability, with P80 showing a slightly more robust increase in translocation compared to Hn-33. In contrast, when the same experiment was conducted in HT-29 cells (Figure 6B), the difference in DrBoNT/A transport with and without P80 or Hn-33 was not statistically significant (p > 0.05). This suggests that the effects of P80 and Hn-33 on paracellular transport are more pronounced in Caco-2 cells than in HT-29 cells, which may be attributed to the distinct functional properties and structural differences of these cell lines.

These results support our hypothesis that P80 treatment significantly alters the trafficking of co-incubated marker molecules, particularly in Caco-2 cells. Furthermore, they provide insight into how certain proteins, like P80 and Hn-33, can modulate epithelial permeability, which may be crucial for drug delivery strategies or understanding the mechanisms through which botulinum toxins traverse epithelial barriers.

## Discussion

In this study, the neurotoxin-associated protein P80 was isolated from the BoNT Type E complex using a sequential two-step purification strategy that combined DEAE Sephadex A-50 anion-exchange chromatography with, yielding a highly purified single-component protein fraction. Biophysical characterization of the purified molecule revealed that P80 possesses a predominantly α-helical secondary structure interspersed with regions of random coil, suggesting intrinsic flexibility that may contribute to its biological function. Importantly, cytotoxicity assays demonstrated that P80 did not induce measurable toxicity in intestinal epithelial cells even at a high dose of 300 nM after 24 h of exposure (Figure 3A). This biological profile differs markedly from well-characterized neurotoxin-associated proteins (NAPs) such as Hn-33 from *BoNT/A*, which exhibit hemagglutinin activity and notable sugar-binding properties that mediate cell recognition and can lead to cellular damage. In contrast, P80 was found to lack hemagglutinin activity, indicating its apparent inability to bind glycan structures at the epithelial surface, a feature that may underlie its lower cytotoxic potential.

To explore the functional role of P80 in modulating epithelial permeability, a series of *in vitro* assays were conducted on polarized CaCo-2 monolayers using confocal microscopy, atomic force microscopy (AFM), and permeability/translocation assays. Comparative visualization demonstrated clear differences in the localization and intracellular trafficking of P80 versus Hn-33 (Figure 4), reflecting divergent modes of interaction with the intestinal epithelium. Both proteins induced cytoskeletal remodeling, specifically actin reorganization at tight junction boundaries, though this effect was more moderate and spatially restricted in the case of P80 (Figure 5). Because the actin cytoskeleton forms a structural scaffold for tight junction complexes—linking cytosolic plaque proteins to transmembrane claudins and occludins—localized contraction of actin filaments can generate mechanical tension that “pulls open” junctions, thereby increasing paracellular flux. This junctional relaxation mechanism appeared robust with Hn-33, consistent with previous studies showing its capacity to disrupt epithelial integrity by opening tight and adherens junctions (Fujinaga et al., 2007), while P80 elicited a more regulated and potentially tunable level of junctional remodeling. Although crystal structural studies suggest that Hn-33 does not directly interact with BoNT, biochemical assays have demonstrated a direct association between Hn-33 and BoNT (Sharma and Singh, 1998), indicating that such interactions may occur under physiological conditions despite not being captured in crystallographic analyses. Similarly, our findings show that P-80 directly associates with DrBoNT/A, a nontoxic recombinant derivative of BoNT/A, as evidenced by their co-elution during gel filtration chromatography (data not shown), supporting the existence of a stable protein–protein interaction in solution.

AFM imaging provided nanoscale, topographical confirmation of these structural changes. Treated monolayers exhibited observable separation of adjacent epithelial cells at their intercellular borders (Figure 5A), with the effect being notably pronounced following P80 treatment. This mechanical reorganization correlated strongly with permeability data (Figure 6), wherein translocation assays using co-incubated therapeutic cargo (DrBoNT/A) revealed an increased transport rate in the presence of P80. These results collectively support the hypothesis that P80 acts as a bioenhancer by facilitating the trafficking of co-administered molecules across the epithelial barrier.

Beyond structural remodeling, biochemical analyses indicated that P80 may also exert regulatory effects on the molecular composition of the tight junction complex itself. Specifically, P80 treatment was associated with an increase in phosphorylated claudin-1 levels in CaCo-2 cells (and disruption of TJP protein integrity (data not shown). Claudin-1 phosphorylation at serine, threonine, or tyrosine residues is known to modulate tight junction assembly, barrier strength, and protein-protein interactions. As suggested by classical models of junctional regulation (Pinto da Silva and Kachar, 1982), such post-translational modifications can weaken inter-claudin binding forces, facilitating greater paracellular permeability. Thus, P80 appears to enhance barrier transport through a dual mechanism: controlled actin-dependent physical widening of intercellular spaces, and biochemical modulation of tight junction protein networks.

Taken together, this body of evidence establishes P80 as a promising intestinal bioenhancer with a unique mechanistic profile—exhibiting low cytotoxicity, lacking sugar-dependent hemagglutinin activity, and operating through relatively controlled modulation of epithelial barrier function. While Hn-33 serves as a powerful positive control for junction disruption, P80 may offer a more therapeutically favorable balance between efficacy and safety, enabling enhanced delivery of biologics and peptide-based therapeutics across mucosal surfaces.

## Conclusion

The restricted oral bioavailability of many therapeutics continues to pose a major challenge in clinical medicine, driving the need for safe and effective bioenhancers capable of improving intestinal absorption. In this study, we demonstrate that P80 is a promising candidate for such applications. Our findings confirm that P80 is structurally stable—predominantly α-helical—and exhibits no detectable cytotoxicity or hemagglutinin activity, distinguishing it from classical BoNT-associated NAPs such as Hn33. Functionally, P80 significantly enhances paracellular transport across intestinal epithelial layers, enabling translocation of co-incubated marker and therapeutic molecules.

Mechanistically, immunofluorescence and cellular assays suggest that P80 facilitates this enhanced transport by modulating actin filament organization and altering tight junction protein arrangement. While the molecular signaling cascades governing this activity require further elucidation, the observed widening of tight junctions indicates that P80 engages a novel regulatory pathway distinct from previously characterized tight-junction disruptors.

P-80 appears capable of reversibly modulating epithelial barrier permeability and enhancing the translocation of large protein cargos, it represents a promising candidate as a bioenhancer or delivery adjunct for therapeutic proteins and peptides. Such properties suggest potential utility in improving mucosal delivery of biologics. However, translation of P-80 into a targeted delivery platform would require further engineering to achieve tissue specificity, controlled and reversible barrier modulation, cargo conjugation strategies, and minimization of off-target effects, none of which have yet been experimentally established.

Likewise, while P-80 demonstrates promise as a permeation-enhancing carrier molecule, its broader applicability as a universal carrier for nanoparticle- or protein-based therapeutics remains to be determined. Comprehensive evaluation with a diverse range of therapeutic cargos, including proteins, peptides, and nanoparticle formulations, will be necessary to define the scope of its carrier potential. Nevertheless, based on its demonstrated cargo association and epithelial translocation-enhancing properties, the possibility that P-80 could serve as a broadly applicable delivery platform cannot be excluded at this stage.

Collectively, these results identify P80 as a stable, non-toxic bioenhancer capable of modulating epithelial barrier permeability to support improved drug absorption. The unique mechanism of action and promising biological profile of P80 lay the foundation for its future development as part of oral delivery strategies for macromolecular therapeutics, offering the potential to increase systemic availability and broaden clinical applicability of otherwise poorly absorbed drugs.

Although our *in vitro* findings provide evidence for the bioenhancer activity of P-80, *in vivo* experiments are necessary to confirm whether these effects can be reproduced in a complex physiological setting. Factors such as protein stability, enzymatic degradation, mucus interactions, biodistribution, immune responses, and systemic safety may significantly influence the performance of P-80 *in vivo*. Therefore, future studies will be directed toward evaluating the *in vivo* efficacy of P-80 in relevant animal models to assess its ability to enhance cargo delivery under physiological conditions. In addition, these studies will be essential to investigate its pharmacokinetics, tissue distribution, reversibility of barrier modulation, and potential short- and long-term safety. Such *in vivo* validation will provide a more comprehensive understanding of the translational potential of P-80 as a bioenhancer and delivery platform.

## Data Availability

The original contributions presented in the study are included in the article/supplementary material, further inquiries can be directed to the corresponding authors.
